# Editorial: Microbiome associated with plant pathogens, pathogenesis, and their applications in developing sustainable agriculture

**DOI:** 10.3389/fmicb.2024.1423961

**Published:** 2024-05-21

**Authors:** Jian-Wei Guo, Osama Abdalla Abdelshafy Mohamad, Xiaolin Wang, Dilfuza Egamberdieva, Baoyu Tian

**Affiliations:** ^1^College of Agronomy and Life Sciences, Yunnan Urban Agricultural Engineering and Technological Research Center, Kunming University, Kunming, China; ^2^State Key Laboratory of Desert and Oasis Ecology, Xinjiang Institute of Ecology and Geography, Chinese Academy of Sciences, Urumqi, China; ^3^College of Agriculture, South China Agricultural University, Guangzhou, China; ^4^Faculty of Biology, National University of Uzbekistan, Tashkent, Uzbekistan; ^5^Fujian Key Laboratory of Developmental and Neural Biology, College of Life Sciences, Fujian Normal University, Fuzhou, China; ^6^Institute of Fundamental and Applied Research, National Research University TIIAME, Tashkent, Uzbekistan; ^7^Department of Environmental Science, Institute of Environmental Studies, Arish University, El-Arish, Egypt

**Keywords:** plant pathology, plant or pathogen associated microbiome, phytopathogenesis, plant-microbe-pahtogen interaction, sustainable agriculture

Plants are susceptible to diverse pathogens and pests, including viral, bacterial, and fungal pathogens, as well as nematodes and other pests. Studies have demonstrated that more than 50,000 parasitic or pathogenic plant diseases are reported worldwide, causing over 40% of total production losses in agriculture in most developing countries (Khaled et al., 2017; Jongman et al., 2020). Phytopathogens and pests pose some of the most devastating threats to productivity and yield, causing destructive plant diseases or damage in both natural and agricultural environments (Egamberdieva et al., 2011; Thynne et al., 2015). Identifying the causal organisms responsible for devastating crop diseases, understanding how they have emerged, and their population epidemiology is of vital importance for enhancing agronomic practice efficiency, ensuring food safety, and developing sustainable agricultural system. Recent advancements in culture-independent techniques, particularly high-throughput sequencing (HTS)-based whole genomic and metagenomic analysis, have provided us with novel insights into the pathogenesis of plant diseases. This shift has moved the focus from individual microbes to a pathobiome paradigm, or systems-based plant pathology (Ray et al., 2020; Mannaa and Seo, 2021). Research focusing on the widely accepted pathogen-disease hypothesis has led to many breakthroughs, such as the identification of multi-origin pathogens of plant known or novel diseases (Ray et al., 2020). Wang X. et al. (2022) and Wang Y. et al. (2022) discovered the soybean stay-green associated virus through the multi-omic integration of bacterial, fungal and viral microbiome data. Furthermore, this disease was associated with a dramatic increase microbial loads and dysbiosis of the bacterial microbiota in seeds. Furthermore, community analysis has allowed us to observe the pathogen-disease paradigm in detail, considering not only the pathogens capable of growing *in vitro* culture, but also systems-based plant pathology, where communities and their interactions are considered rather than individual organisms (Jongman et al., 2020; Mannaa and Seo, 2021). Phytopathogenesis is not only the outcome of the interactions between pathogens or pests and their host plants and but also the result of interactions with the microbiomes associated with plant hosts and pathogens. Among these, the microbiome associated with the plant host and pathogen plays a central role in regulating plant health and pathogen infection.

The plant microbiome including endophytic, epiphytic, and rhizosphere microbiomes, has shown great potential in agricultural systems for desired agronomic and ecological functions (Carrion et al., 2019; Shen et al., 2024). Plants can recruit specific microbial taxa to adapt to environmental stress. In return, the microbiome can activate genes related to host plants to obtain required nourishment and indirectly improve the fitness of host plants under environmental stress such as nitrogen deficiency and pathogen invasion. For example, the abundance of the genus *Massilia* showed a negative correlation with soil nitrogen content, revealing *Massilia* as a key taxon that drives root microbiota assembly under nitrogen deficiency. Additionally, the inoculation of *Massilia* enhanced both root and shoot growth of *zm00001d048945* mutants under conditions of nitrogen deficiency (He et al., 2024). Microbiome-related genes such as *NRT1.1B* in rice, have been shown to effectively improve the establishment of the rice root microbiome and nitrogen use efficiency (Zhang et al., 2019). Accordingly, the plant-associated microbiome has been regarded as the plant's second genome (Mendes et al., 2011; Berendsen et al., 2012; Wang et al., 2021). The collective genome of the host organism and its symbiotic microbiome is considered as the “hologenome,” jointly facilitating the development of new varieties with higher yields and increased pathogen resistance (Li et al., 2024).

However, microbiomes do not always interact with host plants as favorable partners. Growing evidence suggests that disease occurrence in plants is often accompanied by changes in the associated microbiome (Tian et al., 2015; Kwak et al., 2018). Microbiomes may also detrimentally affect plant health or promote parasitism or pathogenicity by establishing symbiotic or mutual relationships with pathogens or pests, thereby facilitating the occurrence of plant diseases (Ray et al., 2020; Mannaa and Seo, 2021). Li et al. (2023) suggested that the community structure and assembly of root endophytic microbiota were significantly affected by root-knot nematodes (RKN) parasitism, revealing an association of nitrogen-fixing bacteria with RKN infection. Liu et al. (2024) identified that non-pathogenic *Pseudomonas syringae* could induce a “cry for help” response from the host plant, leading to the assembly of growth-promoting and disease-suppressing rhizomicrobiomes. Understanding interactions among plants, pathogens and microbiomes will help us develop novel plant disease control strategies (Xun et al., 2022; Zhou et al., 2022; Li et al., 2023).

We organized this topic, “*Microbiome associated with plant pathogens, pathogenesis, and their applications in developing sustainable agriculture*” to consolidate recent findings and perspectives encompassing a broad spectrum of plant pathology, ranging from individual microbes to pathobiomes, or systems-based plant pathology. This includes research on the identification of plant pathogens, pathogenesis, microbiomes associated with plants and pathogens, nematodes or pests, as well as biological control of plant diseases or the enhancement of healthy plant microbiomes to foster sustainable agriculture, among other aspects. We extend our gratitude to all the authors and reviewers for their valuable contributions to this topic, which comprised of two mini-reviews and fourteen original research articles.

Chen et al. conducted a review on the occurrence, corresponding pathogens and geographical distribution of fungal diseases on *Camellia oleifera*. They proposed an integrated approach to control these fungal diseases, which includes establishing monitoring and forecasting systems, breeding disease-resistant varieties, implementing biological control techniques, and employing a rational application of chemical fungicides. Zhou et al. provided a summary detailing the strategies employed by various hemibiotrophic pathogens interacted with host immune receptors to activate plant immunity. They highlighted the significant role of the plasma membrane in plant immune responses, as well as the current obstacles and potential future research directions.

Wang et al. discovered that leaf crinkle of *Lycium barbarum* was caused by a novel cytorhabdovirus named as “goji cytorhabdovirus A (GCVA)” identified using HTS. The virus possesses a linear, negative sense single-stranded RNA genome of 14,812 nucleotides and encodes six open reading frames. Then two phylogenetic analyses of the L protein and N protein demonstrate that GCVA should be classified as a new species in the genus *Cytorhabdovirus*. Additionally, the RT-PCR detection and RT-LAMP assay were effectively developed to detect the new virus. Liu C. et al. reported that *Fusarium oxysporum* was identified by the morphological and molecular characteristics as the causal agent of bulbous rot on *Lilium davidii* var. *willmottiae*. They found that the most suitable temperature for mycelial growth was 28°C, while the optimal relative humidity for spore production was 55%. Additionally, cinnamon essential oil exhibited superior antifungal efficacy against the pathogen.

Guo Z. et al. and Liu F. et al. unveiled pathogenesis and pathogenicity using different pathogen-disease plant models, separately from pathogen and host plant. Guo Z. et al. obtained three mutants (*Cm699, Cm854*, and *Cm1078*) of *Colletotrichum magnum*, which caused fruit rot on watermelon. They found that the mutant completely lost pathogenicity, producing significantly fewer or no conidia than the WT strain. Additionally, six potential virulence genes related to pathogenicity and sporulation were identified. In another study, the transcriptome and proteome of the highly resistant cultivar “Renong No. 1” and cultivar “Keitt” in response to *Xanthomonas citri* pv. *mangiferaeindicae* infection at different stages were compared, resulting in 14,397 differentially expressed genes (DEGs) and 3,438 differentially expressed proteins (DEPs). Finally, three hub genes/proteins (SAG113, SRK2A, and ABCB1), which play an important role in plant hormone signal transduction, were identified (Liu F. et al.).

Systems-based plant pathology has demonstrated that pathogenesis and parasitism in plants have significant effects on the taxonomy, composition, and assembly of pathogen-disease associated microbial communities. Diseased strawberries had a higher microbial diversity than those of healthy ones in both the root surface and root rhizosphere soils. The relative abundances of the genus *Colletotrichum* in diseased roots increased 5–6 times more than those in healthy roots, implicating *Colletotrichum* as the major pathogen in strawberry fields. Additionally, strawberry root rot resulted in the decreased microbial interaction network stability, and more endophytic-plant pathogenic and saprophytic groups (Zhang et al.). Tie et al. revealed that the invasion of *Verticillium dahliae* on tomato plants might specifically recruit beneficial microbiomes, including *Pseudomonas*. Moreover, resistant cultivars had a more complex bacterial community network relationship than susceptible cultivars. Cao et al. found that RKN-infected tobacco exhibited a richer and more diverse rhizosphere soil bacterial community rather than healthy tobacco in most planting areas. Soil pH was the key factor affecting both the microbial composition of tobacco rhizosphere microbiome and the occurrence of RKN disease in the tobacco-growing areas. Zhu et al. showed that the age of the peach trees had a significant impact on the yeast community structure of non-rhizosphere and rhizosphere soils of peach trees. Additionally, soil pH and conductivity were also key factors contributing to changes in soil yeast community structure in the peach orchards.

Application of bio-agents, organic fertilizers and cropping management for developing efficient plant disease control strategies, as well as the improving healthy plant microbiomes, is necessary for achieving sustainable agriculture. Cui et al. isolated antagonistic bacteria from the V-Ti magnetite tailings and elucidated their control effects on *Corynespora cassiicola*, which was the pathogen of kiwifruit brown spot. Among 136 bacterial strains isolated, 18 strains demonstrated inhibitory activity against *C*. *cassiicola*. Notably, *Bacillus* sp. KT10 exhibited effective control of kiwifruit brown spot in both pot and field experiments. The application of *Bacillus amyloliquefaciens* Ba13 with RNAi modulation enhanced plant growth and host resistance to tomato yellow leaf curl virus (TYLCV) by enhancing RNAi-related gene expression and increasing viral genome methylation levels, suggesting a novel efficient tool and strategy to control viral plant diseases using the RNAi-based antiviral system in the presence of microbial agents. The inoculation of *B. amyloliquefaciens* Ba13 in tomato plants with viral infection promoted a 15.4% increase in plant height, and the relative abundance of the TYLCV gene in tomato leaves decreased by 70.1%, compared with those of non-inoculated controls (Guo Q. et al.). Arbuscular mycorrhizal fungi (AMF) is another promising microbial agent used in agriculture to enhance plant growth and productivity, and control plant diseases. The bioinoculants of AM *Funneliformis mosseae, Gigaspora gigantea* and *Acaulospora laevis* led to the increased plant development, yield, quality of beetroot and gene expression analysis (ALDH7B4 and ALDH3I1) (Yadav et al.). Besides microbial agent, biofertilizers and cropping management were also among the most efficient and economical agricultural practices for the establishing a sustainable agriculture system. Oyster shell powder and oyster shell powder with organic microbial fertilizer significantly increased soil total nitrogen (16.2% and 59.9%), soil total carbon (25.8% and 27.7%), pH (56.9% and 55.8%), and electrical conductivity (377.5% and 311.7%), facilitated tomato seed germination, and increased the relative abundance of beneficial bacteria like *Massilia, Brevundimonas*, and *Lysobacter*, while decreasing the pathogenic bacteria unidentified_*Burkholderiaceae* (Zheng et al.). Shi et al. investigated the effect of field fertilization treatments on soil microbial communities and plant health at different growth stages of cotton. The results showed that reducing chemical fertilizer combined with the usage of organic fertilizer significantly increased soil available nitrogen and phosphorus, affecting the composition and diversity of bacterial and fungal communities throughout the entire cotton growth period. Evaluating effects of plant cropping systems on soil properties, microbial communities, and crop yield indicated that soybean and maize crop rotation, especially using soybean-maize-maize and maize-soybean-soybean planting systems, could increase soil total organic carbon and nutrients, and promote soybean and maize yield. Moreover, the soybean-maize-maize significantly increased the proportion of some beneficial microorganisms and reduced the soil-borne animal and plant pathogens (Sun et al.).

## Author contributions

J-WG: Writing—original draft. OAAM: Writing—review & editing. XW: Writing—review & editing. DE: Writing—review & editing. BT: Writing—review & editing.
